# Fimepinostat Promotes Apoptosis and Decreases Cytokine Secretion in *NF2*-Related Human Schwannoma Cells

**DOI:** 10.3390/ijms27062636

**Published:** 2026-03-13

**Authors:** Anna Nagel, Ethan W. Hass, Hollie Hayes, Lenna Huelbes, Sofia Oliveira, Haley M. Hardin, Mikhail Marasigan, Eric Nisenbaum, Carly Misztal, Fred F. Telischi, Michael E. Ivan, Xue-Zhong Liu, Olena R. Bracho, Christine T. Dinh, Cristina Fernandez-Valle

**Affiliations:** 1 Burnett School of Biomedical Sciences, College of Medicine, University of Central Florida, Orlando, FL 32816, USA; 2 Department of Otolaryngology, University of Miami Miller School of Medicine, Miami, FL 33136, USA; 3 Department of Neurological Surgery, University of Miami Miller School of Medicine, Miami, FL 33136, USA; 4 Sylvester Comprehensive Cancer Center, Miami, FL 33136, USA

**Keywords:** Neurofibromatosis Type 2, schwannoma, fimepinostat, CUDC-907, HDAC, histone deacetylase inhibitor, NF-κB, tumor necrosis factor receptor

## Abstract

There is no approved drug therapy for schwannomas associated with *NF2*-related schwannomatosis (*NF2*-SWN). Neither life-saving surgical resection or radiation are curative and can compound the debilitating neurological effects of the schwannomas. We previously identified fimepinostat, a dual histone deacetylase (HDAC)/phosphoinositide-3 kinase (PI3K) inhibitor, as a promising drug candidate with pro-apoptotic effects on *NF2*-related schwannomas. This preclinical study used the pharmaceutical formulation of fimepinostat to confirm its efficacy in schwannomas and identify pro-apoptotic signaling pathways. Fimepinostat was tested in human schwannoma model cells, patient-derived primary vestibular and non-vestibular schwannoma cells, and in a sciatic nerve allograft model. The signaling pathways leading to caspase-3-dependent apoptosis were elucidated using immune assays, flow cytometry, imaging, proteome, and acetylome analysis. Acute exposure to fimepinostat led to p21-dependent cell cycle inhibition, upregulation of tumor necrosis factor-related apoptosis-inducing ligand receptor 2 (TRAIL R2), and downregulation of tumor necrosis factor receptor 1 (TNFR1), Yes-associated protein (YAP), and inhibitors of apoptosis. Moreover, fimepinostat downregulated cytokine and chemokine secretion increased by merlin loss in schwannoma cells. Fimepinostat is a promising new drug intervention for *NF2*-SWN patients with the potential to promote tumor regression.

## Simple Summary

This study focuses on the action of fimepinostat, a dual histone deacetylase (HDAC)/phosphosphoinositide-3 kinase (PI3K) inhibitor in *NF2*-related schwannomatosis. Decreased tumor sized in a mouse sciatic nerve allograft model accompanied with mechanistic studies on human model cell lines suggests that HDAC inhibition might be a feasible antitumor therapy in *NF2*-related schwannomatosis.

## 1. Introduction

*NF2*-related schwannomatosis (*NF2*-SWN) is caused by pathogenic mutations in the *NF2* gene, which encodes the tumor suppressor protein merlin [1,2,3]. Individuals with *NF2*-SWN are predisposed to the development of nervous system tumors, including schwannomas, meningiomas, and ependymomas. Although overall tumor burden and disease severity vary among individuals with *NF2*-SWN, nearly all patients develop bilateral vestibular schwannomas (VSs) on the vestibulocochlear nerve that eventually cause hearing loss, tinnitus, and imbalance [4]. Treatment options are limited to surgical intervention, radiation, or enrollment in clinical trials. Surgery is non-curative and may compromise the adjacent facial nerve, resulting in permanent hearing loss and facial paralysis. Radiation carries the risk of malignant transformation of the tumors in *NF2*-SWN patients [5]. Despite many efforts, there is still no Food and Drug Administration (FDA)-approved pharmacological treatment for *NF2*-SWN. To date, clinical trials have reported modest success at inhibiting tumor growth and/or improving hearing [6,7,8]. These therapies are typically effective for six months to three years before tumor regrowth or cumulative adverse effects end treatment. The limited success rate of these clinical trials highlights the need for further research into therapeutic strategies for *NF2*-SWN [9].

We previously reported that fimepinostat (also known as CUDC-907), a dual histone deacetylase (HDAC)and phosphoinositide-3 kinase (PI3K) inhibitor, selectively promoted cell death in human schwannoma models and patient VS cells and reduced the growth of sciatic nerve allografts by 44% compared to vehicle-treated mice [10,11]. Significantly, fimepinostat induced caspase-dependent apoptosis in human schwannoma model cells and five patient VS cell lines. PI3K signaling is regulated by merlin and is activated in *NF2*-related schwannomas [12,13]. Multiple preclinical studies have evaluated PI3K inhibitors alone and in combination with p21-activated kinase (PAK) and focal adhesion kinase (FAK) inhibitors for *NF2*-SWN, demonstrating significant cytostatic effects; however, none reported cell death in human schwannoma models [14,15,16,17].

HDACs are universal epigenetic regulators that control gene expression associated with cell proliferation, differentiation, and death [18]. HDAC1 and HDAC2 are crucial regulators of myelination in Schwann cells, controlling the expression of beta-catenin and SRY-box transcription factor 10 (Sox10), respectively [19]. Other pan-HDAC inhibitors, panobinostat and AR-42, have been studied in *NF2*-SWN models [11,14,20,21]. AR-42 is currently under evaluation for *NF2*-related meningiomas in a phase II clinical trial (NCT05130866). Fimepinostat has been evaluated in clinical trials for triple-negative breast cancer/ovarian cancer (NCT02307240) and B-cell lymphomas (NCT01742988) and is in a clinical trial for pediatric and young adult patients with solid brain tumors (NCT03893487) [22].

Here, we confirmed the efficacy of the pharmaceutical formulation of fimepinostat in eleven additional patient-derived vestibular schwannoma cells and in the mouse sciatic nerve allograft model. Moreover, we identified the early changes in protein acetylation caused by fimepinostat and mapped the altered signal transduction pathways associated with schwannoma cell death.

## 2. Results

### 2.1. Fimepinostat Induces Cell Death In Vitro and In Vivo NF2 Schwannomatosis Models

**Figure 1:**
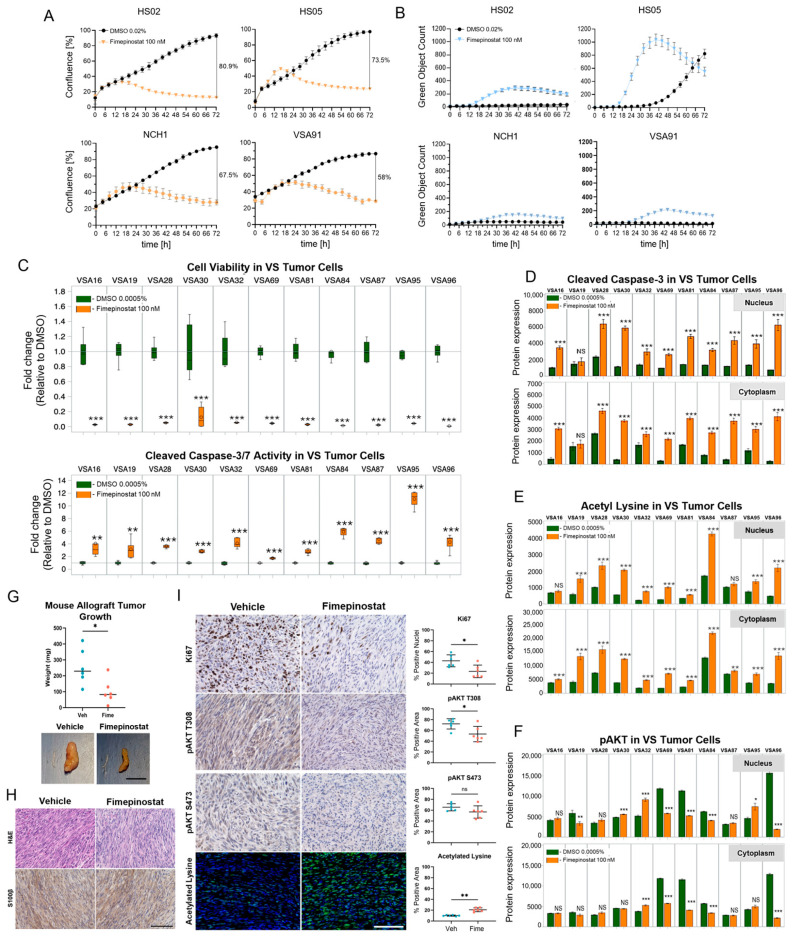
**Fimepinostat Induces Cell Death in vitro and Slows Growth of Sciatic Nerve Allografts.** (**A**). Confluence curves for human schwannoma model cells (HS02 and HS05) and patient-derived schwannoma (NCH1 and VSA91) cells treated with 100 nM fimepinostat and dimethyl sulfoxide (DMSO) over 96 h. (**B**). Green object counts indicating caspase-3/7 cleavage over time during the same treatment as panel A. (**C**). Cell viability fold change (**top**) and cleaved caspase-3/7 signal (**bottom**) in 11 patient-derived vestibular schwannoma (VS) cell samples after 72 h treatment with 0.0005% DMSO or 100 nM fimepinostat (** *p* < 0.01, and *** *p* < 0.001). (**D**–**F**). Quantitative summary of 11 primary VS (including 4 *NF2*-related) treated for 24 h with fimepinostat (100 nM) versus vehicle (0.0005% DMSO) by nucleus and cytoplasm (* *p* < 0.05, ** *p* < 0.01, and *** *p* < 0.001) presented for cleaved caspase-3 (**D**), acetyl lysine (**E**), and p-protein kinase B (AKT) (**F**). (**G**). Murine sciatic nerve allograft weights following 21-day treatment (5 days/week) with 75 mg/kg fimepinostat and vehicle (*p* = 0.026). Representative sciatic nerve schwannoma allografts with contralateral control nerves are shown for each condition. Scale bar = 1 cm. (**H**). Representative hematoxylin and eosin (H&E) stains and S100β (brown) immunohistochemistry for fimepinostat- and vehicle-treated allografts. Scale bar = 50 µm. (**I**). Representative immunohistochemistry and quantification for conditions indicated. (* *p* < 0.05 and ** *p* < 0.01, scale bar = 50 µm, brown colorimetric and green immunofluorescence (IF) indicate positive signal, blue stains nuclei).

The efficacies of research-grade fimepinostat (Selleckchem, S2759, Houston TX, USA) and its pharmaceutical formulation (Curis, Inc.) were compared; both had similar 50% growth inhibitory (GI50) concentrations in human schwannoma model cells (6.09 and 0.74 nM for HS02 and 341 and 357 pM for HS05, respectively; Supplementary Figure S1A). Cell confluence dynamics and caspase cleavage were assessed in HS02 and HS05 cells, *NF2*-SWN patient-derived spinal schwannoma cells (NCH1), and *NF2*-SWN patient-derived VS cells (VSA91). Fimepinostat inhibited cell proliferation within 18 to 24 h of addition in all cell lines. After 72 h, the confluence reduction in fimepinostat-treated cells versus dimethyl sulfoxide (DMSO) was 80.9% for HS02 (*p* < 0.0001), 73.5% for HS05 (*p* < 0.0001), 67.5% for NCH1 (*p* < 0.0001), and 58.0% for VSA91 (*p* < 0.0001, Figure 1A). All cells increased the cleaved caspase 3/7 (CC-3/7) signal, with peaks between 36 and 48 h of fimepinostat treatment (Figure 1B). In patient-derived VS cells, 100 nM fimepinostat significantly reduced cell viability by 72 h in eleven samples (*p* < 0.001, Figure 1C), four of which are from *NF2*-SWN patients (VSA19, VSA29, VSA30, and VSA69). CC-3/7 was significantly elevated by 36 h (** *p* < 0.01 and *** *p* < 0.001, Figure 1C) and remained elevated at 48 h (Supplementary Figure S1B). After 24 h of 100 nM fimepinostat treatment, cleaved caspase-3 (CC-3) was detected by immunofluorescence in 10 of 11 VS primary cells (91%) and in three of four *NF2*-related VSs (75%) when compared to DMSO-treated cells (Figure 1D, *** *p* < 0.001). Concomitantly, acetylated lysine levels increased in all VS cells in the cytoplasm and in most nuclei (9 of 11, 82%), consistent with robust inhibition of histone deacetylase activity (Figure 1E, ** *p* < 0.01 and *** *p* < 0.001). By contrast, phospho-protein kinase B (AKT) decreased in only 5/11 primary VS cells (45%, Figure 1F, * *p* < 0.05, ** *p* < 0.01, and *** *p* < 0.001). In vivo efficacy of fimepinostat was evaluated using sciatic nerve allografts treated with vehicle or 75 mg/kg fimepinostat (Curis) over 3 weeks following a 5-day on, 2-day off dosing schedule. Fimepinostat-treated mice had a 61% reduction in average graft weight compared to vehicle-treated controls (*p* < 0.025, Figure 1G). Both treatment groups displayed similar tumor histopathology and expressed the schwannoma marker, S100β (Figure 1H). Allograft immunohistochemistry revealed lower Ki-67 positivity in fimepinostat-treated allografts (24% vs. 43% positive nuclei; *p* < 0.05; Figure 1I). Phosphorylation of Akt (T308) decreased from 72% to 52% positive area (*p* < 0.05) and nuclear acetylated lysine increased from 10% to 21% positive area (*p* < 0.01) in fimepinostat-treated versus vehicle-treated grafts.

### 2.2. Fimepinostat Targets HDAC2 and Increases Acetylation of Non-Histone Proteins in Schwannoma Cells

**Figure 2:**
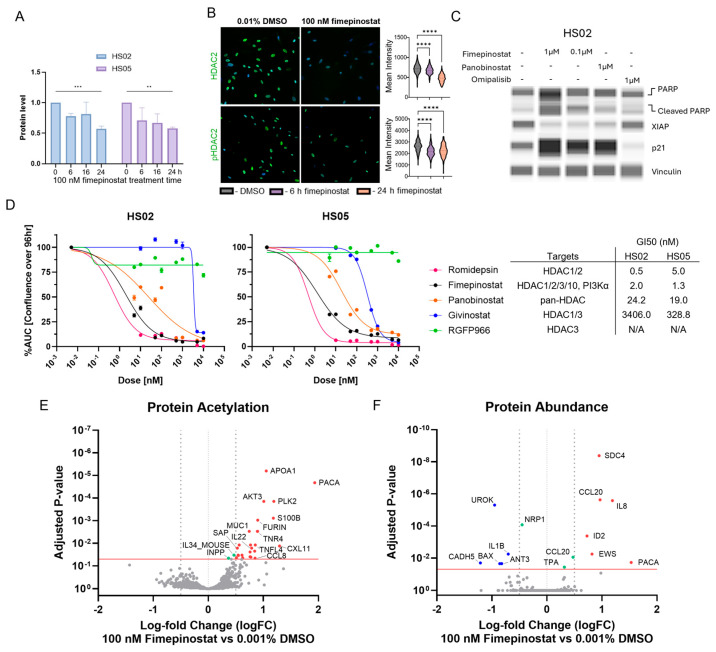
**Fimepinostat Targets HDAC2 and Increases Acetylation of Non-histone Protein.** (**A**). Quantitation of capillary-based immunoblots for histone deacetylase 2 (HDAC2) in HS02 and HS05 cells treated with 100 nM fimepinostat for 6, 16, and 24 h. Normalized to 24 h 0.001% DMSO levels (*** *p* = 0.0008 and ** *p* = 0.0011). (**B**). Representative immunostaining images of HS02 cells treated with 100 nM fimepinostat for 24 h. Nuclear intensity quantification of HS02 at 6 and 24 h 100 nM fimepinostat and 24 h 0.001% DMSO treatment (**** *p* < 0.0001, green—positive staining, blue—nuclei). (**C**). Capillary-based immunoblots for HS02 cells treated with 1 and 0.1 µM fimepinostat, 1 µM panobinostat (pan-HDAC inhibitor), and 1 µM omipalisib (PI3K inhibitor) for 24 h testing cell death-related protein expression. (**D**). Area under the curve (AUC) analysis of cell confluence with indicated treatment. Proteomics analysis of HS05 cells treated with 100 nM fimepinostat for 6 h for (**E**) non-histone protein acetylation and (**F**) protein abundance (log fold change cut off > ±0.5 indicated by gray dotted lines and adjusted *p*-value cutoff < 0.05 indicated by red horizontal line). Red proteins—higher acetylation/abundance in fimepinostat-treated cells, blue proteins—lower acetylation/abundance in fimepinostat-treated cells, green proteins—*p*-value < 0.05 but did not meet log fold change cutoffs (small effect).

Fimepinostat exhibits selectivity in inhibiting HDACs, specifically HDAC 1, 2, 3, and 10, with some activity towards other HDACs [10]. HDAC2 expression was the highest in HS02 and HS05 cells (Supplementary Figure S2A). Downregulation of HDAC2 was observed after 24 h of 100 nM fimepinostat (Selleckchem) treatment in both cell lines (*** *p* = 0.0008 and ** *p* = 0.0011; Figure 2A, Supplementary Figure S2A). Quantification of HDAC2 and phosphorylated HDAC2 (pHDAC2) nuclear staining intensity revealed a significant decrease in both after 6 h of fimepinostat treatment, with pHDAC2 showing a more substantial decrease at that time point (**** *p* < 0.0001, Figure 2B). Moreover, the majority of the eleven primary VS tumor chunks tested expressed HDAC2 and pHDAC2 (Supplementary Figure S2B). Fimepinostat and another HDAC inhibitor, panobinostat, but not the PI3K inhibitor omipalisib, induced Poly (ADP-ribose) polymerase (PARP) cleavage, X-linked inhibitor of apoptosis (XIAP) downregulation, and p21 (Waf1/Cip1) upregulation in HS05 cells, indicating that the pro-apoptotic and anti-proliferative effects of fimepinostat are related to its ability to inhibit HDACs, not PI3K, at the doses tested (Figure 2C and Supplementary Figure S2C). Compared with other HDAC inhibitors, fimepinostat was one of the most potent in inducing schwannoma model cell death (GI50 = 2.0 and 1.3 nM in HS02 and HS05, respectively; Figure 2D). Only romidepsin, a specific HDAC1/2 inhibitor [23,24], had a ten-fold lower GI50 than fimepinostat (GI50 = 0.5 and 5.0 nM for HS02 and HS05, respectively; Figure 2D). This demonstrates that targeting HDAC2 has the greatest anti-proliferative and pro-apoptotic effect on schwannoma cells. Panobinostat and givinostat, two broad-spectrum HDAC inhibitors, had lower efficacy, and the HDAC3-specific GFRP966 was ineffective (panobinostat GI50 = 24.2 and 19.0 nM; givinostat GI50 = 3406.0 and 328.8 nM for HS02 and HS05 respectively; Figure 2D). Overall, the data support HDAC2 as the major target of fimepinostat, associated with the induction of cell death.

To explore early changes in non-histone protein acetylation, abundance, and phosphorylation, HS05 cells treated for 6 h with 100 nM fimepinostat (Curis, Inc.) were assessed with the scioDiscover protein microarrays (Sciomics GmbH) (Figure 2E and Supplementary Figure S2D). A total of 25 proteins were significantly differentially acetylated with acute treatment. Apolipoprotein APOA1, adenylate cyclase-activating peptide (PACA), and polo-like kinase 2 (PLK2) were the three most significantly acetylated (*p* = 6.34 × 10^−6^, 2.09 × 10^−5^, 1.39 × 10^−4^, respectively; Figure 2E). Other proteins with significantly increased acetylation included AKT3, S100B, furin, prosaposin (PSAP), C-C motif chemokine ligand 8 (CCL-8), C-X-C motif chemokine ligand 11 (CXCL-11), interleukin 22 (IL-22), interleukin 4 (IL-4), and tumor necrosis factor superfamily member 4 (TNFSF4/TNFL4) (Figure 2E). The abundance of 11 proteins was significantly higher in fimepinostat-treated than in vehicle-treated cells, including PACA, syndecan-4 (SDC4), the transcriptional regulators EWS and ID2, and the cytokines interleukin 8 (IL-8) and CCL-20. Proteins significantly decreased in abundance with fimepinostat treatment were IL-1B, antithrombin III, Bax, urokinase (UROK), and cadherin 5 (*p* < 0.006, Figure 2F and Supplementary Figure S2D). Both PACA abundance and acetylation were among the most significantly upregulated by 6 h of fimepinostat treatment (*p* = 2.09 × 10^−5^, logFC = 1.532; Figure 2E,F). However, no effects on proliferation or modulation of fimepinostat response was observed in cells treated with PAC-1 and transforming growth factor β (TGFβ) receptor agonists (PACAP 1-38 and recombinant TGFβ) and antagonists (PACAP 6-38 and LY2109761; Supplementary Figure S2D,E).

### 2.3. Fimepinostat Induces Apoptosis by Downregulating Tumor Necrosis Factor Receptor 1 (TNFR1) and Inhibitors of Apoptosis (IAPs)

**Figure 3:**
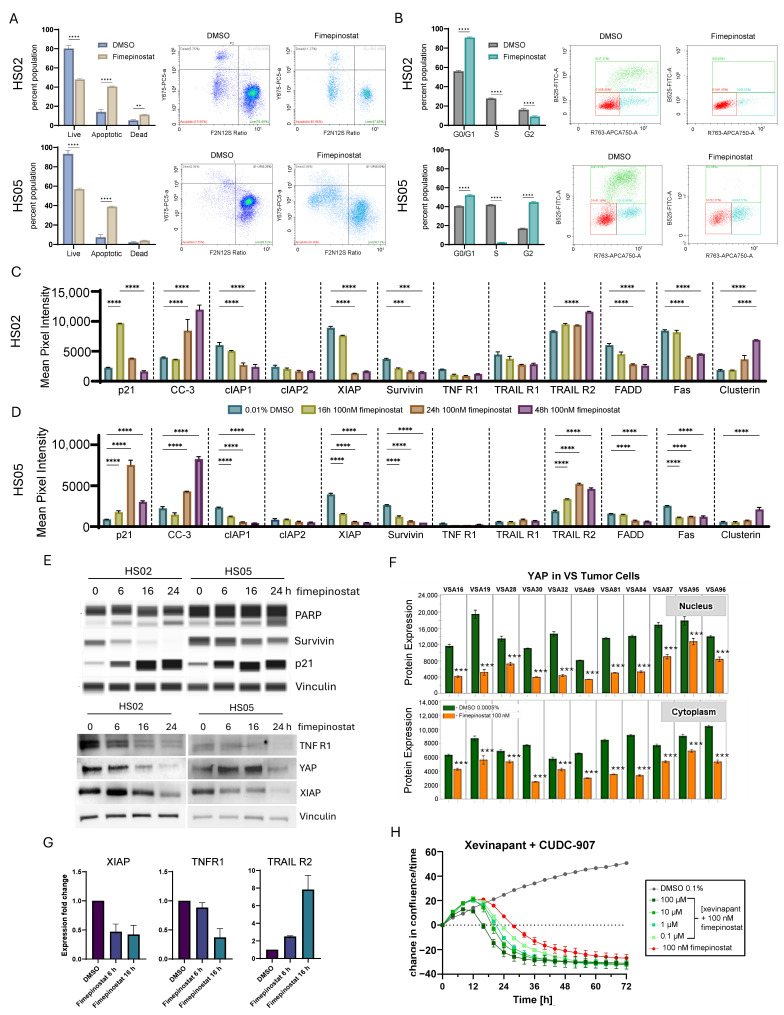
**Fimepinostat Promotes Apoptosis of Human Schwannoma Cells by Downregulating IAPs and TNFR1 and Upregulating TRAIL R2**. Representative flow cytometry plots of HS02 and HS05 cells treated with 100 nM fimepinostat or 0.0125% DMSO for 36 h, showing (**A**) apoptosis and (**B**) cell cycle distribution (Mean ± SD, ** *p* < 0.005, **** *p* < 0.0001). Human XL Apoptosis Array analysis of (**C**) HS02 and (**D**) HS05 cells treated with fimepinostat for 16, 24, and 48 h (Mean ± SD, *** *p* < 0.0005 and **** *p* < 0.0001). (**E**). Immunoblots of HS02 and HS05 cells treated with 100 nM fimepinostat for 6, 16, and 24 h. (**F**). Quantitative result of immunofluorescent staining for yes-associated protein (YAP) of 11 VS cell lines treated with 100 nM fimepinostat for 24 h (*** *p* < 0.001). (**G**). RT-qPCR results for X-linked inhibitor of apoptosis (XIAP), tumor necrosis factor receptor 1 (TNFR1), and TNF-related apoptosis-inducing ligand (TRAIL R2) in 100 nM fimepinostat-treated HS02 cells for 6 and 16 h. (**H**). HS02 cells treated with 100 nM CUDC-907 plus 0.1, 1, 10, and 100 µM xevinapant over 72 h and normalized to starting confluence.

Flow cytometry analysis of HS02 and HS05 treated with 100 nM fimepinostat (Selleckchem) for 36 h revealed an increase in the apoptotic cell population (14.1% to 40.6%, *p* < 0.0001, and 7.5% to 38.9%, *p* < 0.0001, respectively; Figure 3A). HS02 cells also significantly increased in the dead cell population (5.3% to 11.3%, *p* = 0.003; Figure 3A). Cell cycle analysis showed a significant decrease in S-phase populations in both HS02 and HS05 cell lines (27.9%to 0.02%, *p* < 0.0001, and 42% to 2.3%, *p* < 0.0001, respectively; Figure 3B). HS02 cells were arrested at the G0/G1 phase of the cell cycle (55.9 to 95.9%, *p* < 0.0001; Figure 3B), whereas HS05 cells primarily arrested at the G2/M phase (16.9% to 44.7%, *p* < 0.0001; Figure 3B) with an increase in G0/G1 population as well (40.6% to 52.0%, *p* < 0.0001; Figure 3B). This difference could reflect the immortalized nature of HS05 cells. Apoptosis- related protein arrays of HS02 and HS05 cell lysates revealed increased p21 protein by 16 h of fimepinostat treatment in HS02 and 24 h in HS05 (*p* < 0.0001, Figure 3C,D); p21 upregulation was followed by an increase in cleaved caspase 3 (CC-3) starting at 24 h, which persisted at 48 h (*p* < 0.0001, Figure 3C,D). The inhibitors of apoptosis (IAPs) decreased by 16 h for HS05 and 24 h for HS02 and included cellular inhibitor of apoptosis 1 (cIAP1), XIAP, and survivin (*p* < 0.0001, Figure 3C,D). Additionally, upregulation of tumor necrosis factor-related apoptosis-inducing ligand receptor 2 (TRAIL R2) and clusterin, and downregulation of Fas-associated death domain (FADD) and Fas, were observed in both cell lines, with no significant changes in TNFR1 (*p* < 0.0001, Figure 3C,D). Capillary-based immunoblotting confirmed downregulation of survivin and upregulation of p21 by 16 h of fimepinostat treatment in both cell lines (Figure 3E). Increased PARP cleavage at 24 h of fimepinostat treatment confirmed CC-3-dependent apoptosis induction (Figure 3E and Supplementary Figure S3B). Traditional Western blotting revealed downregulation of tumor necrosis factor receptor 1 (TNFR1), yes-associated protein (YAP), and XIAP by 16 h of fimepinostat treatment (Figure 3E). In 11 VS primary cell lines, YAP expression was also significantly reduced in both nuclear and cytoplasmic compartments across all tumors (11/11, 100%; Figure 3F; *** *p* < 0.001). We tested whether XIAP, TNFR1, and TRAIL R2 expressions were transcriptionally controlled using reverse transcription-quantitative polymerase chain reaction (RT-qPCR) of fimepinostat-treated samples. XIAP and TNFR1 gene expression were downregulated at 16 h of the treatment, whereas TRAIL R2 gene expression was upregulated (Figure 3G). To further investigate the role of IAP inhibition in fimepinostat-induced apoptosis, HS02 cells were treated with IAP inhibitor xevinapant in combination with 100 nM fimepinostat over 72 h. The addition of xevinapant significantly shifted the induction of apoptosis in HS02 cells from 24 h to 16 h (Figure 3H).

### 2.4. Fimepinostat Corrects Merlin Deficiency-Related Cytokine Response and Attenuates MCP-1 Secretion

**Figure 4:**
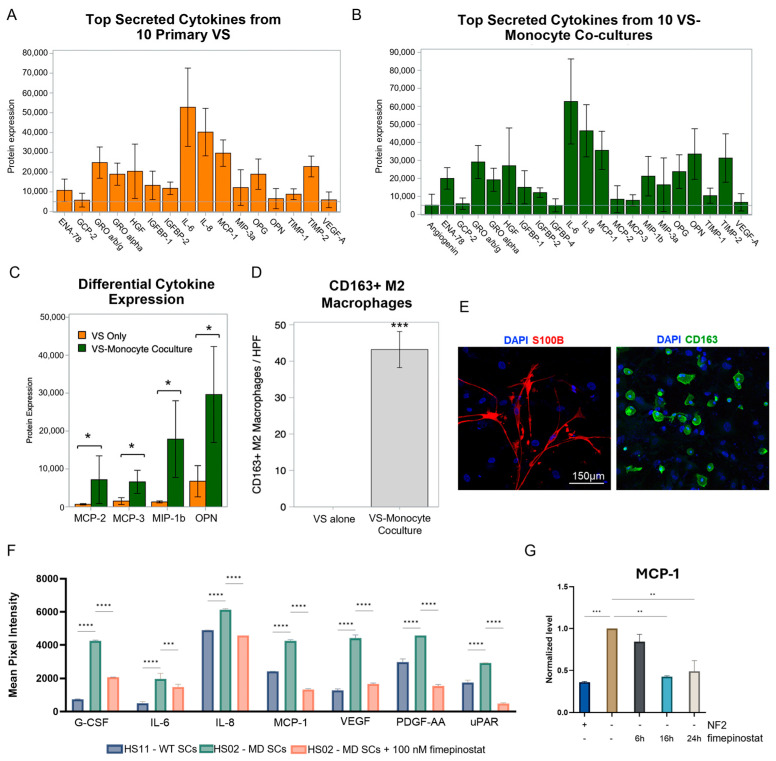
**Fimepinostat Corrects Loss of Merlin-related Cytokine Expression and Decreases MCP-1 Secretion.** (**A**,**B**). Cytokine array analysis of 10 patient-derived vestibular schwannoma (VS) samples showed IL-6, IL-8, and MCP-1 as the most highly expressed in VS cells alone and VS–monocyte cocultures. (**C**). Addition of monocytes to VS cultures significantly increased secretion of MCP-2, MCP-3, macrophage inflammatory protein-1β (MIP-1b), and osteopontin (OPN) (* *p* < 0.05). (**D**,**E**). Immunocytochemistry for CD163+ M2 macrophages (green) and S100B+ VS cells (red), nuclei are stained in blue. (**F**). Cytokine array analysis of HS11 (WT) and HS02 (MD) untreated and treated with 100 nM fimepinostat for 24 h (Mean ± SD, *** *p* < 0.0005 and **** *p*< 0.0001). (**G**). MCP-1 ELISA of HS11 and HS02 cells untreated and treated with 100 nM fimepinostat at 6, 16, and 24 h (Mean ± SD, ** *p* < 0.005 and *** *p* < 0.0005).

We analyzed 10 additional primary VS cells and their cytokine secretion, finding that interleukin 6 (IL-6), IL-8, and monocyte chemoattractant protein-1 (MCP-1) were most highly expressed across all samples tested (Figure 4A). Moreover, conditioned media from 10 VS–monocyte cocultures consistently demonstrated the top secreted cytokines to be IL-6, IL-8, and MCP-1 (Figure 4B). When compared to VS alone, VS–monocyte cocultures also secreted significantly higher levels of MCP-2, MCP-3, macrophage inflammatory protein 3 beta (MIP3β), and osteopontin (OPN) (Figure 4C), which are cytokines critical for monocyte polarization to macrophages and the development of tumor-associated macrophages (TAMs) [25,26]. Monocytes also polarized to M2-subtype CD163+ macrophages in VS–monocyte cocultures, demonstrating the impact of VS and associated secreted cytokines on the tumor immune microenvironment (Figure 4D, E). Because the immune component is of high importance in schwannoma tumors, cytokine secretion was tested in wild-type HS11 cells, merlin-deficient HS02 cells, and HS02 cells treated with 100 nM fimepinostat for 24 h. Granulocyte colony-stimulating factor (G-CSF), IL-6, IL-8, MCP-1, vascular endothelial growth factor (VEGF), platelet-derived growth factor AA (PDGF-AA), and urokinase plasminogen activator surface receptor (uPAR) secretion increased with merlin deficiency (HS02 versus HS11, **** *p* < 0.0001) and decreased in HS02s when treated with fimepinostat (*** *p* < 0.0005, **** *p* < 0.0001, Figure 4F, Supplementary Figure S4). ELISA confirmed MCP-1 secretion upregulation in HS02 vs. HS11 cells (*** *p* < 0.0005, Figure 4G) and MCP-1 downregulation in HS02 cell lines at 6, 16, and 24 h of fimepinostat treatment (** *p* < 0.005, Figure 4G).

## 3. Discussion

*NF2*-related schwannomas pose a significant challenge for patients and clinicians. Their unpredictable and slow but persistent growth within the nervous system creates hesitancy in early surgical intervention. Instead, patients and providers opt for sequential trials with small-molecule inhibitors targeting merlin-dependent proliferation pathways. However, no small-molecule inhibitor has been shown to promote tumor regression or achieve sustained tumor volume control. Over the past three decades, much attention has been given to understanding how merlin regulates proliferation pathways, with comparatively little attention to delineating merlin’s role in cell survival.

### 3.1. Fimepinostat Targets HDAC2 Activity in Human Schwannoma Cells

Fimepinostat is the only small molecule among others evaluated by this group that induces cell death in human schwannoma model cell lines, an in vivo mouse sciatic nerve allograft model, and all patient-derived vestibular and non-vestibular schwannoma cells evaluated. At 100 nM, fimepinostat induces caspase 3/7 cleavage, cell cycle arrest at 24 h, and an increase in the apoptotic cell count by 36 h. Comparative dose–response sensitivity assays, immunofluorescence, and immunoblots identify HDAC2 as the likely critical target of fimepinostat. Only romidepsin, a specific HDAC class I inhibitor, had a lower GI50 than fimepinostat. In contrast, the pan-HDAC inhibitors panobinostat and givinostat were less effective, and the HDAC3-specific inhibitor GFRP966 was ineffective. Thus, HDAC2 is a potential target for *NF2*-SWN tumor response to fimepinostat. Studies in nerve development and regeneration in mice have revealed that HDAC2 in Schwann cells promotes the transcriptional program of myelination in synergy with Sox10 [19]. Moreover, HDAC2 is the most highly expressed HDAC in acute and chronic nerve injury in young and aged mice [27]. Chen et al. reported that HDAC1/2-mediated acetylation of NF-κB is crucial for the developmental switch controlling myelination in Schwann cells [28]. Mice lacking both HDAC1 and HDAC2 had severe myelination defects, and individual knock-out (KO) of HDAC1 or HDAC2 induced overexpression of the other. However, fimepinostat-driven downregulation of HDAC2 did not induce HDAC1 overexpression in human schwannoma cell lines. It is possible that, in the absence of merlin or axons, Schwann cells do not utilize HDAC2 to induce NF-κB-dependent myelin gene expression, but rather to induce expression of injury-associated cytokines and inhibitors of apoptosis. Recently, others have hypothesized that schwannoma cells are in a “repair cell” state [29,30]. We found that the loss of merlin in normal human Schwann cells promotes expression of a “repair-like” phenotype [31].

### 3.2. Fimepinostat Downregulates Inhibitors of Apoptosis, TNFR1, and Upregulates TRAIL R2

We observed a strong upregulation of p21 levels as early as 6 h post-fimepinostat treatment, suggesting that p21 is a major contributor to schwannoma cell cycle arrest. It has been reported that p21 induction can be p53-dependent [32]. However, fimepinostat did not alter p53 levels, as assessed by apoptosis immune arrays (Figure S3A). Other reports demonstrated that p21 induction can be independent of p53 and plays an active role in TNF-induced apoptosis via death receptor signaling [33,34]. Indeed, an immune array panel of apoptosis-related proteins showed that both model schwannoma cell lines upregulate TRAIL-R2, with negligible changes in TNFR1 due to the low detection rates. Be-cause of that, TNFR1 protein expression was assessed by Western blotting and RT-qPCR, which revealed TNFR1 downregulation after fimepinostat treatment. Gao et al. proposed that downregulation of TNFR1 or TNFR2 causes Schwann cell apoptosis [35]. A group led by Campana showed that TNFR1 can be selectively sequestered into Schwann cell extracellular vesicles and function as a TNFα decoy, regulating the expression of other cytokines and inhibiting TNFα-induced Schwann cell death. Fimepinostat also reduced YAP levels, which could contribute to cell death signaling. TNF-related apoptosis-inducing ligand (TEAD)auto-palmitoylation inhibitors were shown to be effective in blocking meningioma and schwannoma growth in a *periostin-Cre;Nf2^flox2/flox2^* mouse model with the induction of TUNEL-positive apoptotic cells [36]. However, our previous experience with mouse schwannoma cells indicates significant differences in the responses of mouse and human cells to PI3K inhibition, showing that mouse schwannoma cells are more susceptible to cell death induction than human schwannoma cells [17]. If Hippo pathway inhibition does not induce human schwannoma cell death, but instead has a cytostatic effect, it might be insufficient to reduce tumor size in *NF2*-SWN patients. Indeed, a recently published phase 1/2 trial of the YAP/TEAD inhibitor VT3989 showed that only around 30% of mesotheliomas responded partially, and around 57% remained stable throughout the study [37].

### 3.3. Fimepinostat Attenuates the Cytokine Signature of Merlin-Deficient Schwann Cells

Macrophages participate in nerve repair, pathological demyelination, and tumor growth control [38]. The current theory for schwannoma tumorigenesis posits that merlin-deficient Schwann cells are unable to exit the proliferative repair state following nerve insult and instead generate a perpetual wound response in the form of a schwannoma [39]. In this repair state, Schwann cells secrete pro-inflammatory cytokines and chemokines to recruit immune cells [40]. When comparing growing VS to the static VS, Hannan et al. showed significantly higher infiltration of tumor-associated macrophages (TAMs) with a colinear relationship between TAM infiltration and tumor vascularity [41]. Stankovic et al. showed that schwannoma cells, when compared to Schwann cells, secrete higher concentrations of IL-2 and B-cell activating factor (BAFF) [42]. Indeed, the cytokine protein arrays showed that macrophage-chemoattractant proteins (G-CSF and MCP-1) are significantly upregulated in merlin-deficient cells and downregulated by fimepinostat treatment. MCP-1 is a major macrophage chemoattractant secreted by repair Schwann cells to promote inflammation and clear myelin debris during injury [43]. Nisenbaum et al. showed that cystic fluid from cystic VS contains high levels of MCP-1 when compared to adjacent cerebrospinal fluid [44]. When analyzed by enzyme-linked immunosorbent assay (ELISA), decreased levels of secreted MCP-1 were observed as soon as 16 h after fimepinostat (100 nM) treatment. We suspect that NF-kB-driven gene expression is impaired by HDAC2 inhibition and TNFR1 downregulation.

In addition to macrophage recruitment, we propose that fimepinostat impairs the schwannoma cells’ capacity to program infiltrating immune cells, particularly TAMs. In the acute phase of nerve injury, infiltrating macrophages are predominantly pro-inflammatory M1 but then transition into a pro-tumorigenic M2 phenotype [45]. Recent spatial proteomic studies confirmed that larger populations of M2 macrophages are found in rapidly growing VSs [46]. Nisenbaum et al. showed that VS tumors with unserviceable hearing had higher levels of the M2 signature [47]. In this study, we also showed that the secreted cytokine profile from 10 primary VSs had a significant impact on the polarization of monocytes into M2 CD163+ macrophages. Our proteomic profiling data show a predominantly M2-inhibitory effect of fimepinostat. Acetylation of M2-polarizing factors like CCL-8, IL-22, IL-4, and IL-34 at 6 h of treatment is presumed to impair their function. Decreased IL-1β abundance after 6 h of fimepinostat treatment is of dual importance, as the cytokine can contribute to both immune cell recruitment [37] and schwannoma cell survival and proliferation [48]. Taken together, the proteome data of fimepinostat-treated human schwannoma cells support a model of diminished macrophage recruitment and enhanced schwannoma cell susceptibility to apoptosis as a dual mechanism for clinical benefit.

### 3.4. HDAC Inhibitors as Therapies for NF2-Related Schwannomatosis

Previous clinical studies of AR-42 in NF2-related vestibular schwannomas and meningiomas (NC0000T01129193 and NCT02282917) demonstrated that HDAC inhibitors are safe in *NF2*-SWN patients [21]. The most common drug-related adverse events (AEs) for fimepinostat are low-grade (Grade 1 and 2) diarrhea, fatigue, and nausea using a once-daily oral administration of 60 mg in a 5-day “on” and 2-day “off” schedule for a 21-day cycle [49]. Phase 1 safety and dose escalation study for fimepinostat in relapsed refractory lymphoma and multiple myeloma reported mostly Grade 1–2 diarrhea, fatigue, and nausea, and Grade 3 diarrhea (5%), thrombocytopenia (16%), and neutropenia (2%) in patients with a median age of 63 and stage III-IV cancer (75%) [50]. In 2015, fimepinostat was granted orphan drug designation for the treatment of patients with diffuse large B-cell lymphoma. Recently, fimepinostat completed a phase 1 trial for children and young adults with brain tumors; it was tolerable but without significant clinical activity (NCT03893487) [51]. In contrast, the recent brigatinib clinical trial in *NF2*-SWN patients reported that 30% of patients experienced severe (Grade 3) AEs, including hypertension, creatine kinase increases, and diarrhea [7].

## 4. Materials and Methods

### 4.1. Cell Culture

Fetal human Schwann cells (HSC) obtained from ScienCell (Carlsbad, CA, USA) were used to generate merlin-deficient HS02 cells as previously described [52]. HS05 cells were generated by knocking out the *NF2* gene with CRISPR/Cas9 *NF2*sg1 (sequence: AAACATCTCGTACAGTGACA, provided by Broad Institute) in wild-type adult Schwann cells immortalized with hTERT and mCdk4 by Dr. M. Wallace at the University of Florida [53]. Primary cell lines were isolated from donated *NF2*-related paraspinal, *NF2*-related vestibular, and sporadic vestibular schwannomas under the approval of the institution’s IRB (protocol numbers for UCF: NCH1: 00001428; VSA91: 00001973; and for UM: 20150637). Informed consent was obtained from all subjects included in the study. Primary cells were isolated as previously described [11] and utilized in passages 1–3. All cell lines were maintained in Complete Schwann Cell Medium (SCM, ScienCell) with 1× Schwann cell growth supplement and 5% fetal bovine serum. Cells were incubated at 5% CO2 at 37 °C. Model cell lines were authenticated by STR profiling (ATCC, Manassas, VA, USA) and routinely tested for merlin depletion and mycoplasma contamination.

### 4.2. Cell Viability and Caspase Cleavage Assays

Ten-point dose–response assays were performed in triplicate using the CellTiter Fluor (Promega, Madison, WI, USA) cell viability assay. Briefly, 5000 cells/well were seeded onto 96-well plates (CellBind, Corning, Corning, NY, USA) and treated the next day for 72 h with fimepinostat (Curis, Lexington, MA, USA, at 10 µM-10 pM). Live-imaging and apoptosis assays were performed as previously described [16]. Briefly, cells were treated in triplicate with 100 nM fimepinostat (Curis) and Caspase 3/7 (CC-3/7) Green Apoptosis Assay Reagent (Sartorius, Gottingen, Germany). Cells were then imaged every 4 h at 20× using the Incucyte S5 Live-Cell Analysis System (Sartorius). Confluence and green signal intensity were analyzed using Incucyte analysis software (version 2024B); statistical significance and plots of experiment triplicates were prepared using Prism 10 Software (GraphPad, version 10.5.0). Patient-derived primary VS cells were seeded at 5000 cells/well in 384-well plates, incubated for 24–48 h (n = 5–6), and treated with vehicle (0.0005% DMSO) or fimepinostat (100 nM). CC-3/7 activity was measured at 36 and 48 h using Caspase-Glo (Promega), and at 72 h for viability using CellTiter-Glo 2.0 (Promega). Relative luminescence units (RLUs) were measured using the Glomax Discover System (Promega). Data were analyzed using SAS Software (v9.4).

### 4.3. Sciatic Nerve Allograft Study

Twenty NOD.Cg-Prkdcscid Il2rgtm1Wjl/SzJ (NSG) immune-deficient mice, evenly distributed by gender and aged 7 weeks, were acquired from Jackson Laboratories (Bar Harbor, ME, USA). Care and use were approved by the University of Central Florida Institutional Animal Care and Usage Committee (IACUC; protocol #202000165). Mouse merlin-deficient SCs (MS01-luc) were engrafted after anesthetization via 2–4% isoflurane inhalation and administration of 3.25 mg/kg extended-release buprenorphine (Ethiqa XR, Fidelis Animal Health, North Brunswick, NJ, USA). A total of 5000 MS01-luc cells in 3 µL phenol red-free Leibowitz-15 media were injected using a 33-gauge Hamilton needle into the sciatic nerve of the right lower limb. Humane endpoints for engrafted mice included tumors that exceeded 20 mm diameter in any direction, became ulcerated, restricted ambulation, or caused abnormal vocalizations. No mice met humane endpoints during this study. Diarrhea was noted in fimepinostat-treated cages, but this was expected and did not exclude any animals from the study. Seven days were allowed for the grafts to establish, after which all mice were imaged using the In Vivo Imaging System (IVIS, Perkin-Elmer, Springfield, IL, USA) to confirm bioluminescent signal emitted from each graft. Engrafted mice were randomized with an even distribution of bioluminescent signal and enrolled into a three-week, five-day on, two-day off protocol for vehicle (30% Captisol, Ligand Pharmaceuticals, Jupiter, FL, USA) or 75 mg/kg fimepinostat (Curis) by oral gavage with no blinding. After three weeks of treatment (15 total doses administered), mice were sacrificed by CO_2_ inhalation, tumors were collected with contralateral control nerves, weighed, and prepared for histology. Tumor weight was the primary endpoint to determine efficacy. Drug target modulation and proliferation index (Ki67) were assessed by IHC as a secondary endpoint. A total of 12 mice (n = 6 per cohort) were evaluable at the end of the study; early losses were attributed to loss during handling, failed engraftment, or uncharacteristically large tumors determined by the Grubb’s outlier test. Tumor weights were compared between vehicle- and fimepinostat-treated mice using a two-tailed Mann–Whitney U test in GraphPad Prism 10.4.2.

### 4.4. Histology and Immunohistochemistry

Standard histology and immunohistochemical (IHC) staining were performed as described [11]. The list of antibodies used is available in the Supplementary Methods Section. All samples were imaged using the Keyence (Osaka, Japan) BZ-X800 microscope, capturing between 5 and 20 regions of interest depending on the size of the tissue sections (40× magnification). All images were analyzed semi-quantitatively using the ImageJ software using the ColourDeconvolution2 plugin. Percent positive nuclei and percent positive area comparisons were performed in GraphPad Prism 10.4.2 using two-tailed Mann–Whitney U tests.

### 4.5. Immunofluorescence

Cells were washed, fixed, and permeabilized. Non-specific binding was blocked using 10% normal goat serum (NGS) in Hank’s Balanced Salt Solution (HBSS). Cells were incubated with primary antibodies for 1 h or overnight at 4 °C, followed by goat anti-rabbit or anti-mouse IgG Alexa Fluor 488 (Invitrogen, Carlsbad, CA, USA) secondary antibodies for 1 h with DAPI counterstain (Invitrogen). Cells were imaged either using a Keyence BZ-X800 microscope or a Molecular Devices (San Jose, CA, USA) ImageXpress Pico microscope, capturing two to four regions of interest per replicate. Quantification was performed using ImageJ. Immunofluorescent staining of VS cells was performed similarly, with overnight incubation at 4 °C using primary antibodies, followed by 2 h of secondary antibody incubation, and nuclear labeling with DAPI (Abcam, Cambridge, UK) for 15 min. Primary antibodies are listed in Supplementary Methods. Images were acquired using an Agilent BioTek (Seattle, WA, USA) Lionheart FX microscope, and imaging parameters were set to negative controls and applied consistently to all experimental wells. Raw images were processed for nuclear and cytoplasmic protein expression (pixel intensity) for 6 regions of interest per replicate, and data were analyzed in GraphPad Prism 10.4.2 using Mann–Whitney U tests with SAS software (v9.4).

### 4.6. Flow Cytometry

Membrane asymmetry and cell cycle flow cytometry assays were performed (n = 3) as previously described [16]. Cells were treated with fimepinostat (Curis) for the time indicated and stained with the Violet Ratiometric Membrane Asymmetry kit (Invitrogen) according to the manufacturer’s instructions. Cells were stained using the LIVE/DEAD Fixable Violet Cell Stain, Click-iT™ Plus EdU AF488, and FxCycle™ Far Red kits (Invitrogen) according to the manufacturer’s instructions. Samples were run using the Cytoflex LX flow cytometer (Beckman Coulter, Brea, CA, USA) and analyzed using CyteExpert (Beckman Coulter). Significance was calculated in GraphPad Prism 10.4.2 using two-way ANOVA with Šidák’s multiple comparisons test.

### 4.7. Proteomics Profiling and Post-Translational Modification Analysis

Three replicates of HS05 cells were treated with 100 nM fimepinostat (Curis) and 0.001% DMSO for 6 h, then washed with PBS and flash frozen with liquid nitrogen before shipping to Sciomics GmbH (Neckargemünd, Germany) for proteomics profiling and post-translational modification analysis. Protein was extracted from each sample in scioExtract buffer and analyzed on two scioDiscover antibody microarrays, measuring 1438 different proteins. Each microarray was incubated with either scioPhosphomix or scioAcetyl detection mixes to measure protein-specific serine/threonine/tyrosine phosphorylation or protein-specific acetylation, respectively. Microarray slides were scanned using a Tecan (Männedorf, Switzerland) PowerScanner, and spot segmentation was performed with GenePix Pro 6.0 (Molecular Devices). Median signal intensities were analyzed using the linear models for microarray data (LIMMA) package of R-Bioconductor. Assessment of differential protein abundance, phosphorylation, and acetylation was conducted using two-sided t-tests with *p*-values adjusted by controlling the false discovery rate according to Benjamini and Hochberg.

### 4.8. Protein Arrays

For the Proteome Profiler Human Apoptosis Array (R&D Systems, Bio-Techne, Minneapolis, MN, USA), HS02 and HS05 cells were treated with 100 nM fimepinostat for the indicated times. Samples were lysed in 1× RIPA buffer, and protein concentration was determined using a detergent compatible (DC) assay (Bio-Rad, Hercules, CA, USA). The array procedure was performed according to the manufacturer’s instructions. For the Human XL Cytokine Array kit (R&D Systems), conditioned media was collected and used to detect cytokine release. All arrays were imaged using the Jess System (Protein Simple, Bio-Techne) and quantified with ImageJ 1.54g. Each antibody was assessed in duplicate and normalized to controls (n = 6). The significance was calculated in GraphPad Prism 10.4.2 by two-way ANOVA with Tukey’s multiple comparisons test.

### 4.9. RT-qPCR

HS02 and HS05 cells were treated as indicated (biological n = 3–4). RNA was extracted using TRIzol and purified with the Direct-zol RNA Miniprep kit (Zymo Research, Tustin, CA, USA). Purity and concentration were analyzed with the Nanodrop 8000. Reverse transcription and amplification were performed using a Qiagen RNA-to-Ct one-step kit on a QuantStudio 3 (Thermo Fisher Scientific, Waltham, MA, USA), with each sample run in triplicate. Housekeeping genes *GAPDH* and *YWAHZ* were used to normalize RNA for quantification using the 2^−∆∆ct^ method, and significance was calculated using a *t*-test.

### 4.10. VS–Monocyte Cocultures

Primary VS cells from 10 VS tumors were plated at 8000 cells/well on 16-well culture slides in SCM at 37 °C and 5% CO_2_ for 24 h. Monocytes were then added at 0 or 2000 monocytes/well (n = 3 wells per condition). Progressive media changes (50%) were performed every 48 to 72 h for 14 days. Conditioned media was collected and processed using the Human Cytokine Array C5 (Raybiotech, Peachtree Corners, GA, USA), per manufacturer’s protocol. All arrays were imaged using the Jess System (Protein Simple, Bio-Techne) and quantified with ImageJ with the microarray plug-in. Results were normalized to 6 positive and 6 negative controls. Cells were washed and fixed with 4% paraformaldehyde. Immunocytochemistry was performed with anti-CD163 primary antibody (1:100, OriGene, TA506383), S100B (1:100, Abcam, ab52642), AlexaFluor-488- and 594-conjugated secondary antibodies (1:500; Invitrogen), and DAPI nuclear stain, as previously described [47]. Confocal images were obtained with a 40× oil immersion lens (Leica SP5 Inverted Microscope). The number of CD163+ M2 macrophages per high-power field was quantified per high power field (HPF) and analyzed with the Mann–Whitney U test using SAS software.

### 4.11. ELISA

ELISA for Human CCL2/MCP-1 (R&D Systems) was used to quantify MCP-1 secreted by cells into the medium. HS02 and HS05 cells were treated with 100 nM fimepinostat for 6, 16, and 24 h; untreated HS11 cells served as *NF2*+ control. Cell culture supernatant was collected and stored at −80 °C until assayed. The assays were performed in duplicates according to the manufacturer’s protocol. The optical density was immediately measured at 450 nm using a Molecular Devices SpectraMax iD5 microplate reader.

### 4.12. Western Blot

Protein extraction and Western blots were performed as described [16]. Briefly, protein was extracted in a RIPA buffer. Protein concentration was determined by a DC assay, and loading was normalized across samples. Blots were imaged and quantified using the Bio-Rad ChemiDoc Imaging System (Bio-Rad, Hercules, CA, USA). Capillary electrophoresis-based Western blot assays were performed using the Jess Simple Western with chemiluminescent detection secondary antibodies (Biotechne, Minneapolis, MN, USA). Quantification was performed using Compass for SW (version 7.0.0) and normalized to housekeeping proteins such as vinculin. All samples were prepared in three to four biological replicates. The list of antibodies is available in Supplemental Methods.

## 5. Conclusions

Our findings highlight the potential of HDAC2 inhibition for the induction of cell death in *NF2*-related schwannoma cells and support the initiation of fimepinostat clinical studies in *NF2*-related SWN.

### Study Limitations

This study explores the effects of fimepinostat on tumor cell survival and its influence on the microenvironment. As such, it does not specifically test the direct involvement of HDAC2 in cell death and cytokine expression. We deduced that HDAC2 is the likely tar-get of fimepinostat based on fimepinostat’s comparable potency with romidepsin, an HDAC1/2-specific inhibitor, and the lack of significant expression of HDAC1 by the schwannoma cells. We previously demonstrated that sensitivity to fimepinostat by VS cells correlates pHDAC2 levels. Another limitation of the study is that it is primarily conducted using in vitro human model and patient derived schwannoma cells. Organoid studies and validation in another animal model would advance the understanding of fimepinostat’s action in schwannomas. However, an indication has yet to be identified for fimepinostat, whereas intravenous romidepsin is FDA approved for peripheral T-cell lymphoma.

## Data Availability

The data sets generated during and/or analyzed during the current study are available from the corresponding author on reasonable request.

## Supplementary Materials

The following supporting information can be downloaded at: https://www.mdpi.com/article/10.3390/ijms27062636/s1.

## Abbreviations

The following abbreviations are used in this manuscript:

NF2-SWN	Neurofibromatosis Type 2-related schwannomatosis
HDAC	Histone deacetylase
PI3K	Phosphoinositide 3-kinase
p21	Cyclin-dependent kinase inhibitor p21
TRAIL R2	TRAIL receptor 2
TNFR1	Tumor necrosis factor receptor 1
YAP	Yes-associated protein 1
VS	Vestibular schwannoma
HS02/HS05	Human schwannoma 02/05 model cell line
HS11	Human Schwann cell, isogenic for HS02
VSA91	Vestibular schwannoma cells (patient derived)
DMSO	Dimethyl sulfoxide
PARP	Poly (ADP-ribose) polymerases
XIAP	X-linked inhibitor of apoptosis
IAP	Inhibitor of Apoptosis
ELISA	Enzyme linked immunosorbant assay
MCP	Monocyte chemoattract protein
IL	Interleukin
