# Supplementary material for: Fimepinostat Promotes Apoptosis and Decreases Cytokine Secretion in NF2-Related Human Schwannoma Cells
Source: Int J Mol Sci. 2026 Mar 13;27(6):2636. doi: 10.3390/ijms27062636 (PMC13026985; doi:10.3390/ijms27062636)
Supplement: Supplementary file 1 [file ijms-27-02636-s001.zip › ijms-4156076-supplementary.pdf]

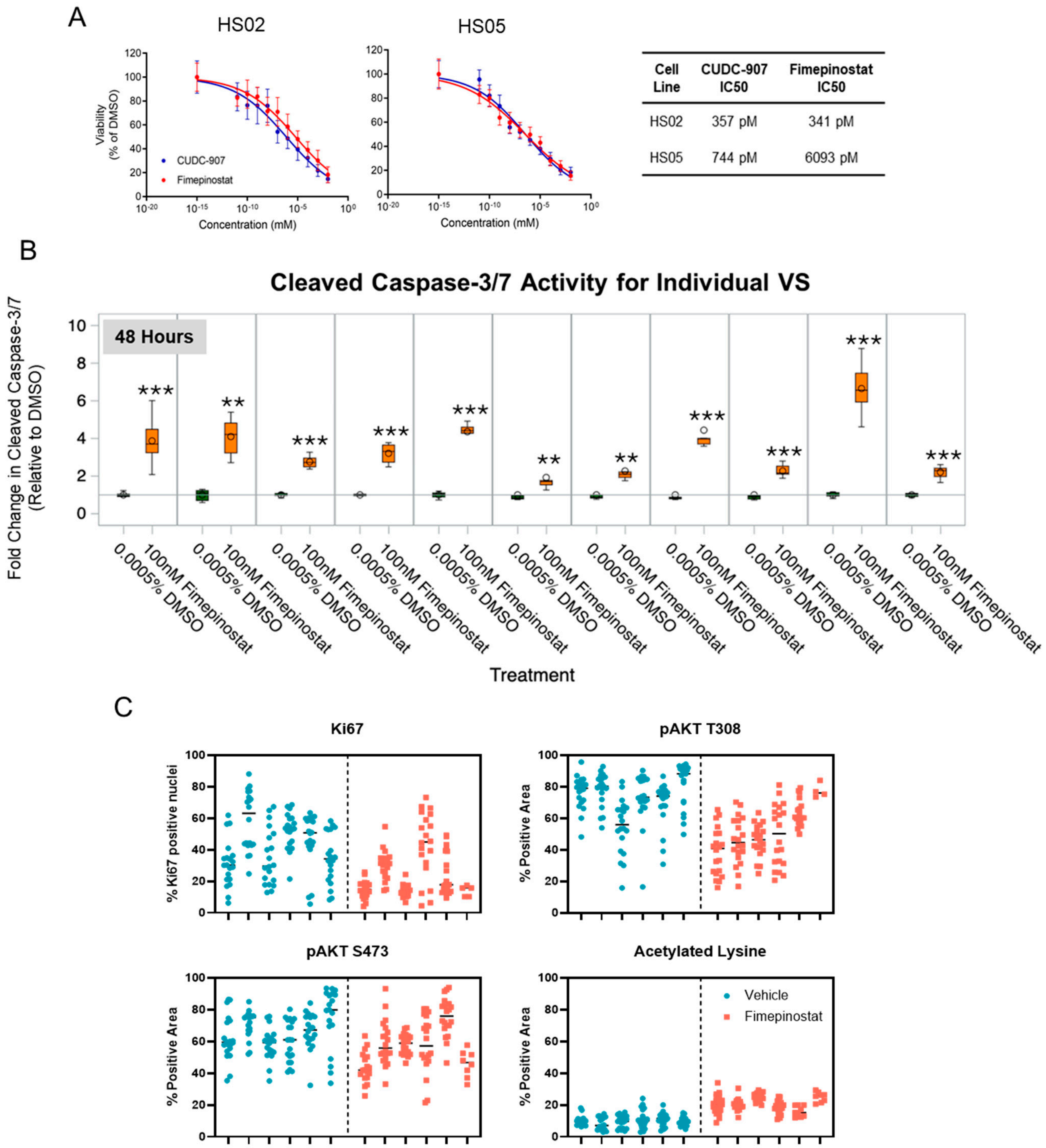

Supplementary Figure 1. A. Cell viability response to research-grade fimepinostat (CUDC-907, Selleckchem) and pharmacologically formulated fimepinostat (fimepinostat, Curis), both formulations had comparable efficacy against human merlin-deficient Schwann cells; **B.** Additional time point assessed during cleaved caspase-3/7 assay for patient-derived vestibular schwannoma cells treated with 100 nM fimepinostat. **C.** All ROI values for immunohistochemistry and immunofluorescence of animal tissue.

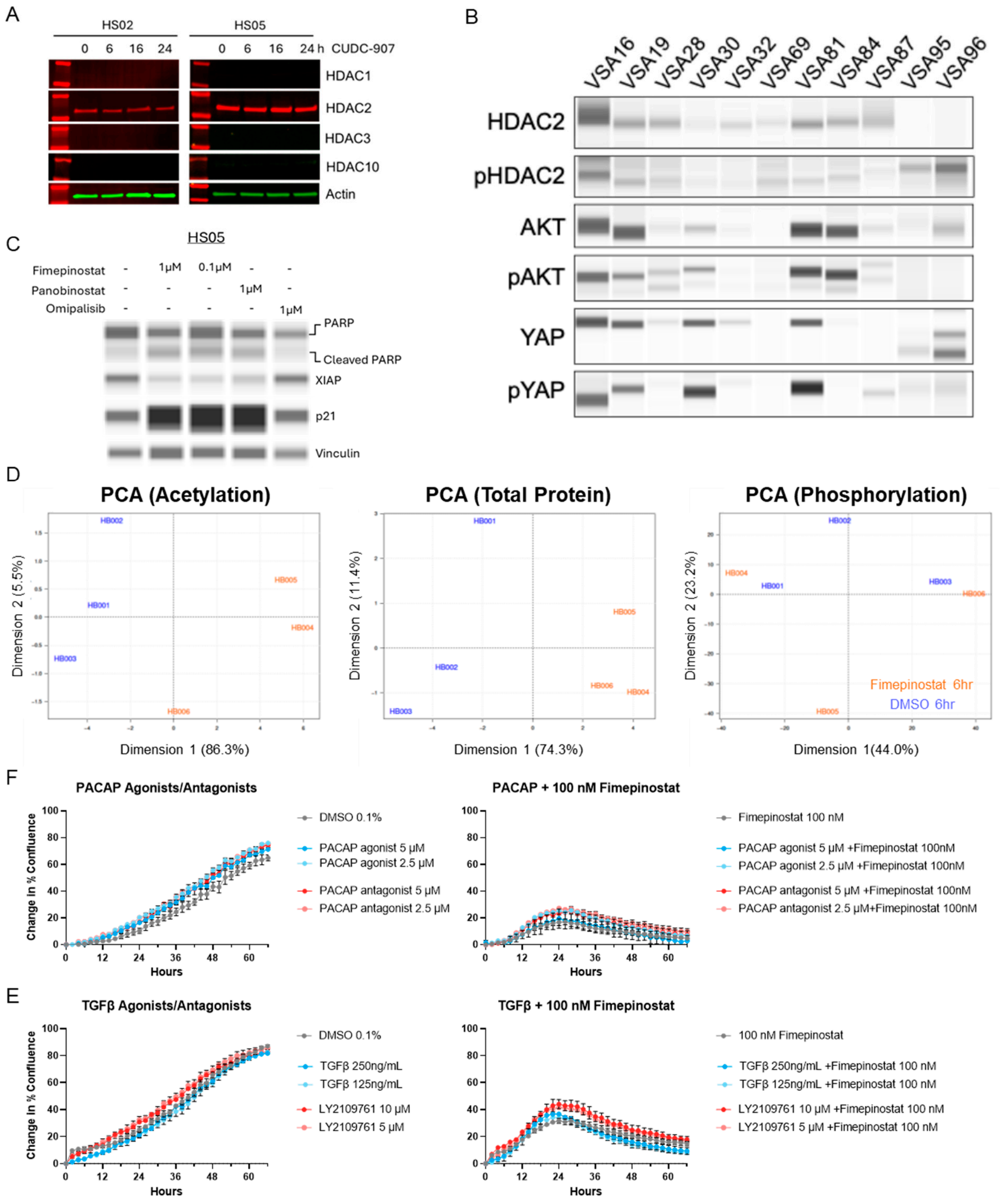

Supplementary Figure 2. **A.** HDAC expression analysis during 100 nM fimepinostat treatment showing no re-upregulation of HDAC1 during the treatment; **B.** Capillary immunoblotting of 11 VS tumor chunks normalized to the total protein abundance; **C.** Jess immmunoblot images of the HS05 cell line treated with fimepinostat, panobinostat and omipalisib confirming findings in the HS02 cell line; **D.** Acetylation and total protein principal component analysis (PCA) of DMSO- and 100 nM fimepinostat-treated HS05 cells for 6 h; **F.** PACAP signaling agonist and antagonist influence on HS05 cell growth with and without 100 nM fimepinostat; **E.** TGFβ signaling agonist and antagonist effect on HS05 cell growth with and without 100 nM fimepinostat.

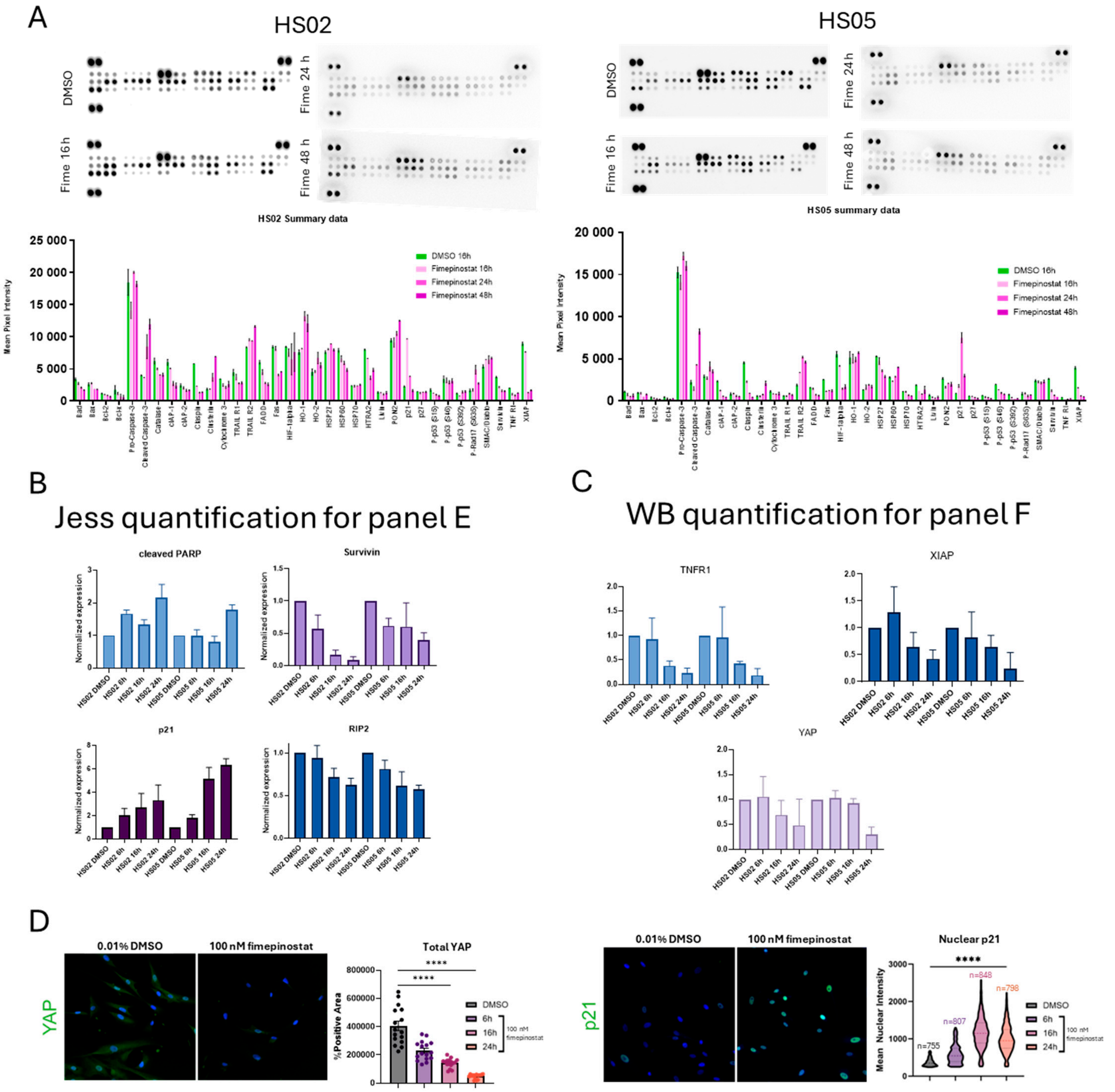

Supplementary Figure 3. **A.** Human apoptosis-related protein array images (R&D Systems) of treated HS02 and HS05 cells with corresponding graphs. **B.** Quantification of Jess western blotting for Figure 3E; **C.** Western blot quantification for Figure 3F; **D.** Immunofluorescence analysis of YAP and p21 in HS02 cells confirming previous western blotting findings that YAP expression is downregulated and p21 expression is upregulated with fimepinostat treatment.

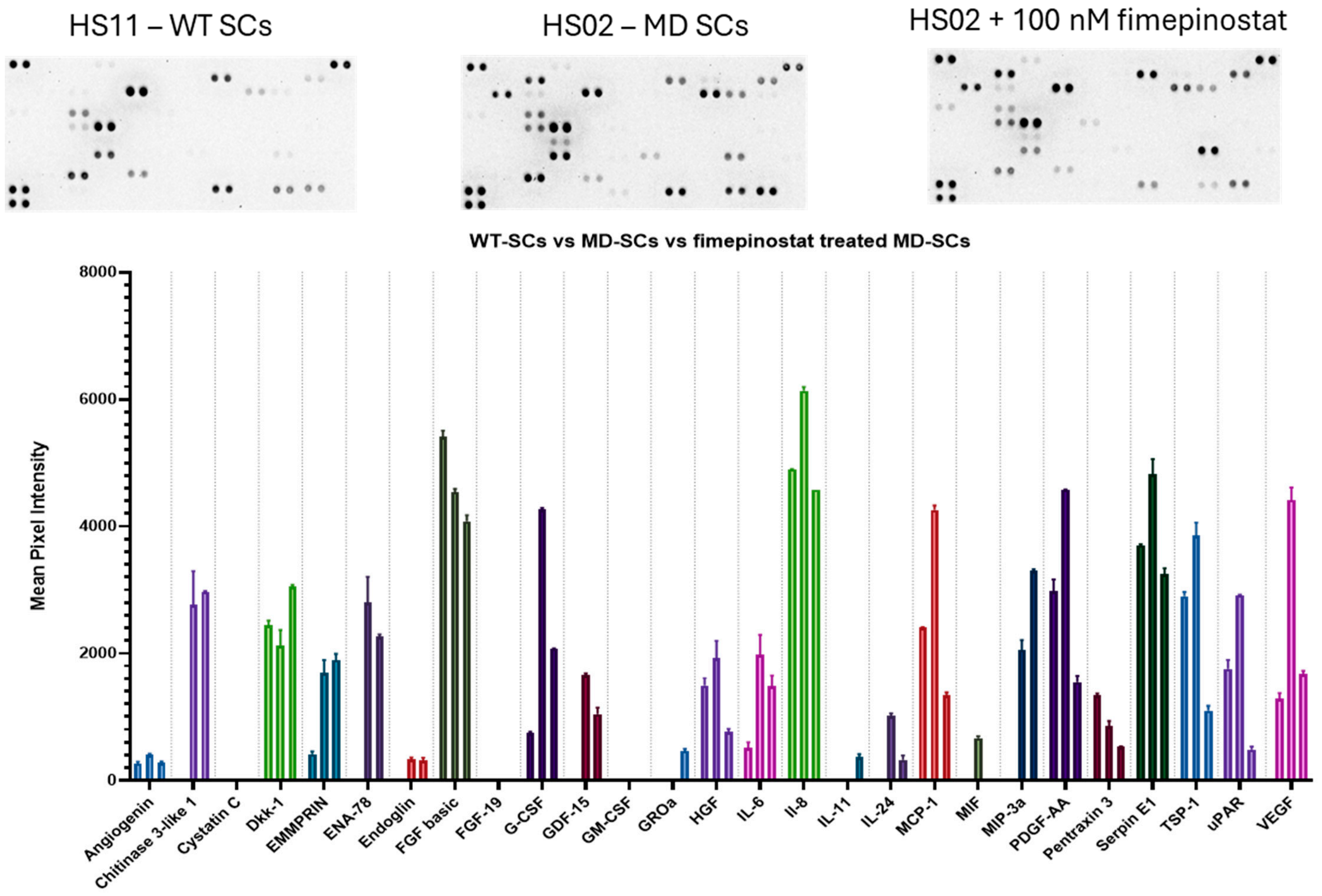

Supplementary Figure 4. Human XL Cytokine protein arrays (R&D Systems) images of HS11 cells, HS02 cells, and HS02 cells treated with 100 nM fimepinostat for 24 h with corresponding quantification . The first bar in the series corresponds to HS11, second bar to HS02 DMSO-treated, and the third to HS02 treated with 100 nM fimepinostat.

Supplementary Methods – antibody and primers lists

Western Blot Primary Antibodies

Antibody	Manufacturer & Catalog	Dilution
p21	Cell Signaling cat. 2947	1:1 000
HDAC2	Cell Signaling cat. 5113	1:1 000
YAP	Cell Signaling cat. 14074	1:1 000
XIAP	Cell Signaling cat. 14334	1:1 000
TNFR1	Cell Signaling cat. 3736	1:800
PARP	Cell Signaling cat. 9542	1:1 000
Survivin	Cell Signaling cat. 2808	1:1 000
Vinculin	Cell Signaling cat. 13901	1:10 000

Immunofluorescence Primary Antibodies

Antibody	Manufacturer & Catalog	Dilution
p21	Cell Signaling cat. 2947	1:600
HDAC2	Cell Signaling cat. 5113	1:200
p-HDAC2 (S394)	Abcam cat. ab75602	1:100
YAP	Cell Signaling cat. 14074	1:200
p-YAP (S127)	Cell Signaling cat. 13008	1:200
Acetylated Lysine	Cell Signaling cat. 9441	1:400
Cleaved Caspase 3 for ICC	Cell Signaling cat. 9661	1:200
p-Akt for ICC	ThermoFisher cat. 701052	1:250
Acetyl Lysine for ICC	ThermoFisher cat. MA1-2021	1:200

Immunohistochemistry Primary Antibodies

Antibody	Manufacturer & Catalog	Dilution
S100b	Novus Bio cat. NBP2-45267	1:200
Neurofilament	BioLegend cat. 837904	1:100
Acetylated Lysine	Cell Signaling cat. 9441	1:400
Ki-67	Abcam cat. 16667	1:200
p-AKT (S473)	Cell Signaling cat. 4060	1:100
p-AKT (T308)	Abcam cat. ab38449	1:200

Primers used for RT-qPCR

Gene	Forward sequence	Reverse sequence
TNFR1	TCA CCG CTT CAG AAA ACC ACC	GGT CCA CTG TGC AAG AAG AGA
TRAIL R2	GCC CCA CAA CAA AAG AGG TC	AGG TCA TTC CAG TGA GTG CTA
XIAP	AAT AGT GCC ACG CAG TCT ACA	CAG ATG GCC TGT CTA AGG CAA
